# Case Report: Isolated homonymous hemianopia after reversal of anticoagulation with andexanet alfa

**DOI:** 10.3389/fneur.2026.1922229

**Published:** 2026-07-30

**Authors:** Paria Malekahmadi, Dina Abdelsalam, Safa Ibrahim, Andrew Go Lee

**Affiliations:** 1 London North West University Healthcare NHS Trust, London, United Kingdom; 2 Houston Methodist Hospital, Houston, TX, United States

**Keywords:** andexanet alfa, apixaban, haemorrhage, homonymous hemianopia, polypectomy

## Abstract

**Purpose:**

Clinical scoring systems have been developed (e.g., CHA2DS2-VASc and HAS-BLED) to aid in the perioperative management of anticoagulation therapy. The decision to continue, modify, or stop anticoagulation prior to surgical procedures requires multidisciplinary input and an individualised plan. Decisions both to continue or reverse anticoagulation can cause visual loss.

**Observations:**

We report the use of andexanet alfa (recombinant inactive factor Xa) as a reversal agent for anticoagulation in atrial fibrillation after surgical bleeding, which resulted in secondary homonymous hemianopsia and ischemic infarct.

**Conclusion and importance:**

Stroke is a major risk factor for atrial fibrillation, and anticoagulation in specific settings is evidence-based. Anticoagulation, however, increases the risk of haemorrhage and may require the use of a reversal agent (e.g., Andexanet alfa) in cases of severe bleeding. Unfortunately, reversal of anticoagulation in this setting can lead to an ischemic stroke and visual loss.

## Introduction

The decision to modify, continue, halt or resume anticoagulation before a surgical procedure requires a multidisciplinary approach and depends on the risk of stroke versus the risk of surgical haemorrhage (1). Direct Oral Anticoagulants (DOACs) are often the preferred treatment to prevent thromboembolism in patients with atrial fibrillation and are considered a safer option compared to warfarin (1). However, in the event of a haemorrhagic episode while on factor Xa inhibitors, andexanet alfa can be administered as a reversal agent (a recombinant form of factor Xa) (1). We report a case of isolated left complete homonymous hemianopia following administration of andexanet alfa for reversal of anticoagulation. Based on our review of the English-language literature, this presentation has not been extensively discussed. Patient consent has been gained.

## Case presentation

**Figure 1:**
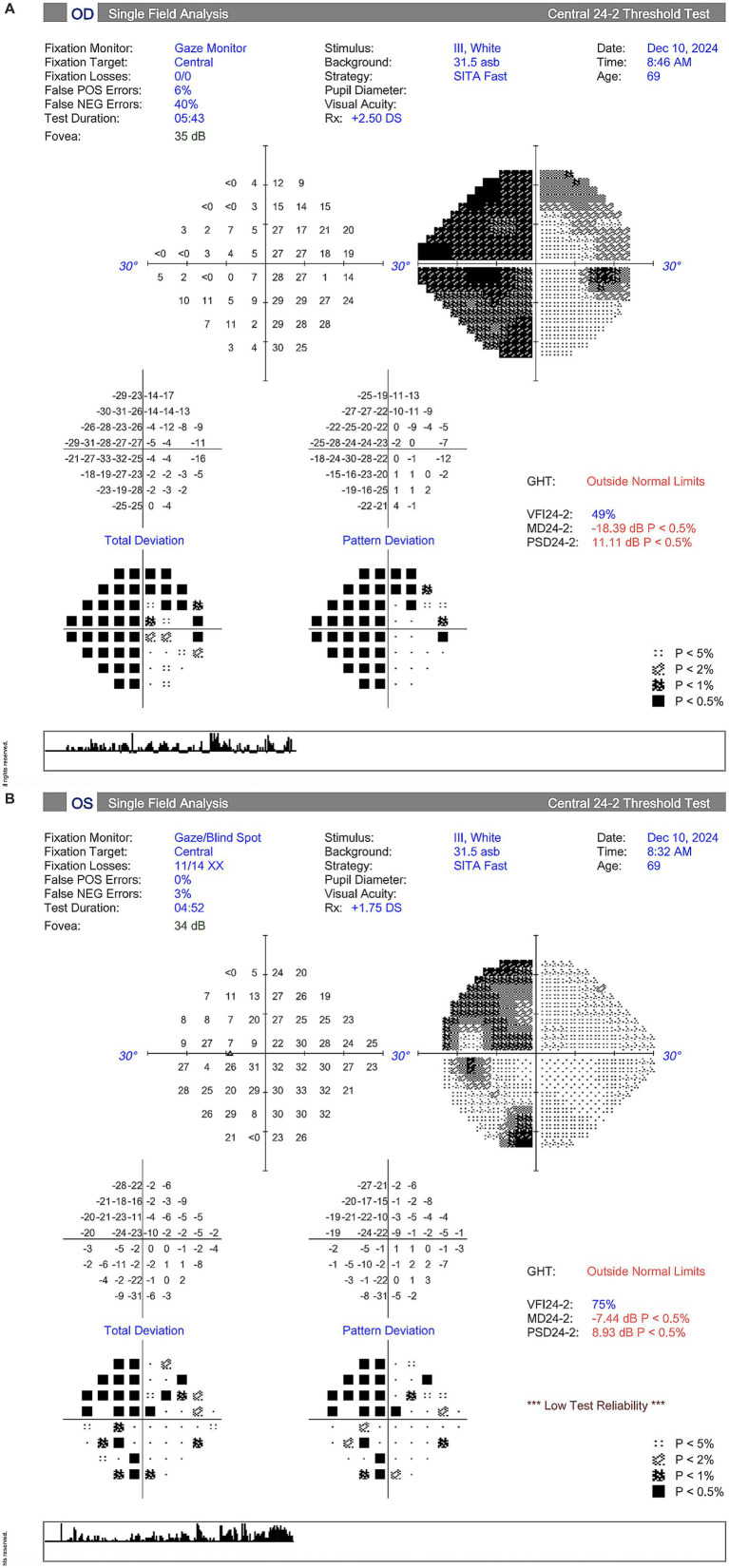
**(A,B)** Humphrey’s 24-2 automated perimetry demonstrates an incongruous left homonymous hemianopia visual field defect.

A 69-year-old male developed a homonymous hemianopia after reversal of chronic DOAC therapy for atrial fibrillation. His CHA₂DS₂-VASc score was 7 prior to treatment. Past medical history included diastolic heart failure, hypertension, obstructive sleep apnoea, diabetes mellitus, angina, and hypercholesterolaemia. He was scheduled for an elective colonoscopy with polypectomy. His medications included amiodarone, metoprolol, furosemide, hydralazine, hydrochlorothiazide, nifedipine, and rosuvastatin (Figure 1).

Anticoagulation therapy with apixaban (Eliquis^®^, Bristol Myers Squibb-Pfizer, United States) 5 mg was stopped electively 2 days before the procedure and then resumed 3 days later. Shortly after colonoscopy with polypectomy, however, the patient developed severe post-procedural anaemia from bleeding. Computed tomography (CT) of the abdomen and pelvis showed an active haemorrhage in the proximal ascending colon. A vascular clip was placed at the bleeding site in the proximal ascending colon to stop the bleeding, and Andexanet alfa (an anti-factor Xa reversal agent) was administered.

**Figure 2:**
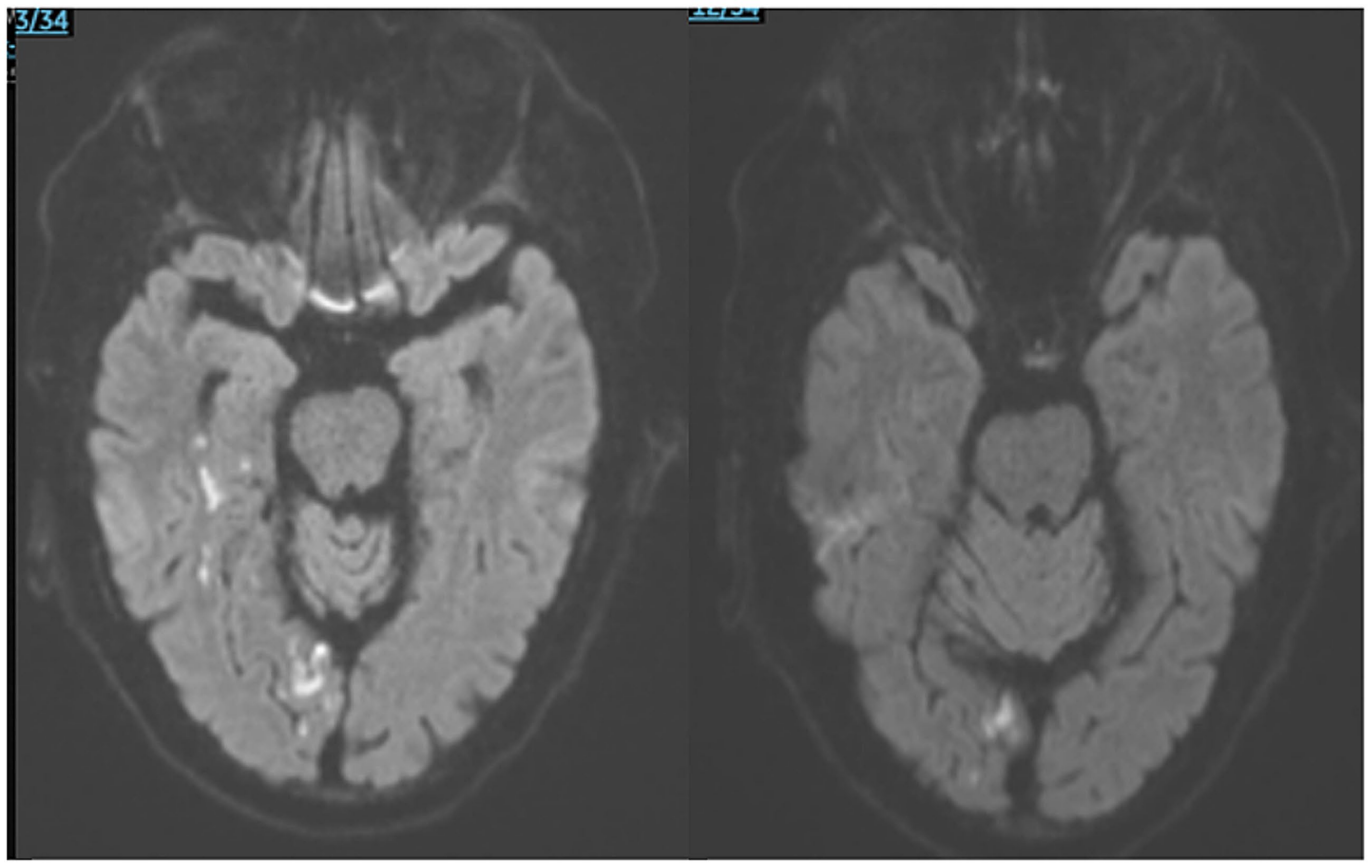
Axial diffusion-weighted magnetic resonance imaging (DWI) demonstrating an acute ischemic infarction involving the right occipital lobe within the posterior cerebral artery territory. The two slices demonstrate the extent of the infarction.

Following administration of Andexanet alfa, the patient developed new-onset headache, dizziness, and vision loss. CT and magnetic resonance imaging (MRI) of the brain demonstrated an acute right posterior cerebral artery infarct, involving the right occipital and posterior right temporal lobes. There were lacunar infarcts in the basal ganglia, pons, and left thalamus (Figure 2). Echocardiogram, carotid Doppler, and CT angiogram of the head and neck were all unremarkable. The neuro-ophthalmic examination revealed a visual acuity of 20/25 in the right eye (OD) and 20/40 in the left eye (OS). Ishihara colour vision test was also normal. The intraocular pressure was 12 and 11 mmHg.

Furthermore, the anterior segment slit lamp examination was normal. Cup-to-disc ratio was 0.5 in each eye and pupils were equal in size and reactive to light with no relative afferent pupillary defect detected. The perimetry (Humphrey visual field 24-2) demonstrated an incongruous homonymous hemianopia. Although occipital infarctions typically produce congruous homonymous visual field defects, multiple lacunar infarcts, as well as the high false-negative rate (40%), may have contributed to the asymmetry.

## Discussion

The British Society of Gastroenterology and European Society of Gastrointestinal Endoscopy guidelines describe polypectomy as a high-risk procedure for secondary bleeding (1). DOACs in this setting are usually discontinued 3 days before these high-risk procedures and then resumed 2 days after. These medications have a rapid onset of action, and an optimal anticoagulation effect can be reached within 3 h of ingesting the first dose (1). Despite guideline-concordant management, this patient developed an acute posterior cerebral artery infarction.

Multiple factors likely contributed to the development of this complication. Firstly, this patient’s CHA₂DS₂-VASc score was 7, highlighting the high risk of thromboembolism. Suspension of apixaban and its resumption 3 days later may have further increased thromboembolic risk. Although the HAS-BLED score in this patient was 3, indicating a significant risk of major haemorrhage, commonly used risk stratification tools do not account for abrupt changes in the coagulation balance after pharmacologic reversal (2).

Andexanet alfa (Andexxa, AstraZeneca, United Kingdom) is a recombinant protein that can reverse the anticoagulant effects of factor Xa (DOAC) inhibitors (e.g., apixaban and rivaroxaban) in patients with potentially life-threatening or severe, uncontrolled bleeding (1). Prior reports of visual complications from andexanet alfa are rare. In one report, an 84-year-old female who was regularly using rivaroxaban 15 mg presented following a head injury. CT scan revealed an acute subdural haematoma; a craniotomy was performed, and andexanet alfa was administered, but led to multiple cerebral infarctions 10 h postoperatively (3).

Andexanet alfa binds and sequesters factor X inhibitors, allowing endogenous factor Xa to regain function (1). In an article critically appraising the Annexa-trial, it was shown that Andexanet alfa can reduce haematoma expansion and achieve a higher haemostatic efficacy (67%) than the control group (53%). Furthermore, 10.3% of patients treated with Andexanet alfa had a thrombotic event at 30 days compared with 5.6% in the control group. 6.5% had an ischaemic stroke compared to 1.5% in the control group. Although haemostasis can be achieved by administering Andexanet alfa, this does not necessarily improve patient outcomes as shown in previous trials conducted. The proposed mechanisms include a transient hypercoagulable state after reversal of anticoagulation.

Andexanet alfa rapidly restores factor Xa activity by sequestering factor Xa inhibitors, which leads to increased thrombin generation. Furthermore, endogenous anticoagulants will be suppressed, leading to a transient prothrombotic state. Although a causal link cannot be confirmed, the timing of events does raise concern (4). In December 2025, AstraZeneca withdrew Andexanet alfa from several markets due to its high risk of fatal thromboembolic events (5).

## Conclusion

This case demonstrates that in the absence of other focal neurological deficits, thrombotic complications such as stroke may initially manifest as visual symptoms only. Use of commonly used stroke and bleeding risk stratification tools, such as CHA₂DS₂-VASc and HAS-BLED, may be limited as they do not adequately account for sudden changes in thrombotic risk following anticoagulation reversal. Therefore, clinicians must maintain a low threshold for diagnosing cerebrovascular events in high-risk patients presenting with acute-onset visual symptoms. This case demonstrates the clinical dilemma of administering anticoagulation therapy (bleeding risk) versus the risk of ischemic thromboembolic stroke in atrial fibrillation. Reversing anticoagulation with Andexanet alfa in the event of severe bleeding restores the baseline thromboembolic risk, calculated using the CHA₂DS₂-VASc score. It can also create a transient prothrombotic state beyond the patient’s baseline risk. When counselling patients about the advantages and disadvantages of reversing anticoagulation, clinicians should be aware of this potential.

## Data Availability

The raw data supporting the conclusions of this article will be made available by the authors, without undue reservation.
